# Patient’s Perceptions of a Centralized Virtual Ward for Remote Patient Monitoring in Primary Care: Qualitative Study

**DOI:** 10.2196/78780

**Published:** 2025-12-01

**Authors:** Alex Jaranka, Gunnar H Nilsson, Terese Stenfors, Maria Hägglund, Panagiotis Papachristou, Marina Taloyan

**Affiliations:** 1 Department of Neurobiology, Care Sciences and Society, Karolinska Institutet, Alfred Nobels Allé 23, Huddinge, 14183, Sweden; 2 Department of Learning, Informatics, Management and Ethics, Karolinska Institutet, Stockholm, Sweden; 3 Department of Women's and Children's Health; Participatory eHealth and Health Data Research Group, Uppsala University, Uppsala, Sweden

**Keywords:** chronic disease, patients perception, primary care, qualitative study, remote patient monitoring, remote sensing technology, telemedicine

## Abstract

**Background:**

Remote patient monitoring (RPM) has the potential to reduce in-clinic visits and promote proactive and preventive care for patients with chronic diseases in primary care. However, a decentralized approach to RPM in a primary health care (PHC) setting has not met stakeholders’ expectations regarding scalability. This study introduces a centralized virtual ward (CVW)–led RPM, utilizing a multidisciplinary team approach to monitor patients with chronic diseases by clinicians who do not belong to the patients' PHC center.

**Objective:**

This study aimed to gain a better understanding of patients’ perceptions of CVW-led RPM for managing chronic diseases in a PHC setting.

**Methods:**

In-depth interviews were conducted with 22 patients with chronic diseases enrolled at a PHC center in Stockholm, Sweden. The RPM project ran between October 2018 and April 2019 and included a total of 395 patients. Interviews followed a semistructured interview guide and were analyzed using qualitative content analysis.

**Results:**

Primary care patients with chronic diseases expressed that their contact with the CVW felt impersonal but at the same time secure and accessible. They noted a lack of coordination and communication between the clinicians of the CVW and their PHC providers. Captured data resulted in 1 overarching theme “Sense of security and accessibility, but impersonal and uncoordinated” based on 5 categories: sense of security, care and self-care, accessibility, quality of care, and communication.

**Conclusions:**

Our findings suggest that by addressing patients’ needs for new organizational routines for patient-caregiver communication, RPM via centralized virtual wards can better realize the potential of this technology.

## Introduction

### Background

The future of health care needs a shift from reactive to proactive and preventive care to reduce the rising global burden of diseases [1]. The World Health Organization defines primary health care (PHC) as “a key process in the health system that supports first-contact, accessible, continued, comprehensive, and coordinated patient-focused care” [2]. A fundamental philosophy of PHC has been to deliver continuity of care to achieve positive patient experiences, greater patient satisfaction, increased treatment adherence, and improved patient outcomes [3].

Globally, PHC is facing numerous challenges, including rising costs, increased public demand for convenience, and technological advances driving personalized health care [4]. New models of health care delivery, including centralized coordination among providers, have been suggested to improve care quality and reduce hospitalizations [5]. Novel digital solutions may enable new forms of interventions and activities that address these challenges [6].

Remote patient monitoring (RPM) is a technology that allows health care providers to monitor, evaluate, and manage patient-reported health data through a remote interface. The collected data are transmitted to the health care provider for clinical review, care management, and patient education [7,8]. RPM enables clinical interventions performed by medical staff who monitor health data, communicate with the patient, and make decisions as a part of the treatment [9].

A plethora of studies indicate that patients with chronic diseases perceive RPM as improving their access to care [10], sense of security [11,12], engagement [13], and disease-specific knowledge [14]. RPM also supports better clinical assessment [15,16], promotes better relationship-based continuity [17], and improves self-management [18]. However, recent studies have identified a lack of competence regarding the use of RPM in clinical care [19]. Also, given the resource constraints and time pressures in PHC, it can be challenging to introduce new tasks such as continuous monitoring of health data.

Virtual wards were first introduced in PHC in 2005, targeting individuals with a very high risk of future emergency hospitalization [20]. The intention was to use multidisciplinary teams and the daily routines of a hospital ward to provide health care in the community [21,22]. A community virtual ward project targeting older adults with complex health care and social care needs resulted in a reduction of emergency department visits and hospital admissions [23]. A centralized health information strategy in the PHC sector has fostered data-driven decision-making, improving the transparency of workflow and preventative care [24].

Combining the virtual ward concept with RPM could potentially offer a more feasible approach to implementing RPM in PHC. The prediction of prior work around CVW and RPM highlights the importance of care coordination and leverages the full range of health care skills within the system to promote patient-centered care [25]. Consequently, it encourages greater patient autonomy and self-management and provides a sense of reassurance [26]. The introduction of CVW models for monitoring patients with COVID-19 during the pandemic may have transformed patients’ experiences of care, making the measurement of patient experience increasingly vital for building a strong, person-centered health care system [27].

### Problem Statement

Despite growing interest among stakeholders, the implementation of RPM in PHC remains limited [28]. Patients support sharing their health data but are concerned about data privacy, security, and management [29]. Clinicians see the benefit of having access to more reported health data but are concerned about increased workload due to the volume of transferred health data [30]. Clinicians also worry that the modernization of health care might lead to a decline in continuity of care [31,32] and reduced patient safety [33]. A major concern among PHC professionals regarding the use of RPM is increased workload due to the transfer of a large amount of data and an increased number of requests from caretakers [34-36]. Health care managers have seen telemonitoring as a means for cost reduction. However, due to increased workload and the cost of technology, managers believed that service centralization would reduce the level of clinician involvement in frontline, resulting in cost savings [32].

A key distinction of the centralized virtual ward (CVW)–led RPM model compared to the traditional in-clinic RPM is that the CVW multidisciplinary care team solely focuses on digital patient interactions based on patient vitals transferred via the RPM platform. The CVW-led RPM team can therefore continuously monitor incoming patient data and interact with the patients without interruptions related to in-clinic workload. This allows the CVW-led RPM model to conduct the monitoring of chronic patients from several clinics, which could therefore address patient and clinician concerns and make the implementation of RPM in PHC more feasible according to health managers' consideration.

The novelty of this study is the examination of the patients’ perception of CVW-led RPM in a PHC setting. To our knowledge, there are no previous publications on this topic.

### Objective

The objective of this study was to explore patients’ perceptions of CVW-led RPM for managing their chronic diseases in a PHC setting.

## Methods

### Study Overview

In this qualitative study, semistructured in-depth interviews were conducted with participants of a digital health intervention, in a Swedish PHC setting. This study is reported in accordance with the Consolidated Criteria for Reporting Qualitative Research checklist [37].

### Centralized Virtual Ward

A private Swedish health care provider, with multiple PHC practices across the country, collaborated with researchers from Karolinska Institutet to establish a CVW-led RPM project at a PHC center in Stockholm. This project was initiated to evaluate the effects of a centralized model of RPM compared to the health care providers’ previous experience of piloting an on-site RPM project, where patients sent their vitals to and communicated with their ordinary clinicians at the PHC.

To establish the CVW-led RPM project, an RPM platform provider was contracted by the health care provider. A specialist doctor in family medicine, 2 specialist district nurses, and an information technology assistant worked part-time at the CVW. All clinical staff at the collaborating PHC were informed about the project. In addition, 2 doctors and 3 nurses from the PHC received training and were given access to the RPM platform for monitoring and communicating with patients during the entire project period.

After the project was launched, the CVW team monitored patient data to detect early signs of deterioration. Elevated vital signs resulted in asynchronous patient contact initiated by the CVW via the platform. Based on the transmitted patient data, medical staff at the CVW were able to personalize treatment plans and make medication adjustments digitally. When signs of deterioration indicated that the participants needed a physical examination, the staff at the patients' PHC center were alerted by the CVW.

The CVW clinicians received training in digital communication with patients but did not conduct any physical examinations during the intervention. Further details of the intervention are available in previously published papers [38,39].

### Remote Patient Monitoring Project

The PHC staff created a list of eligible participants for the project enlisted at the PHC by using a database extraction tool (MedRave) [40], connected to its electronic health records. A flyer with written information about the intervention was mailed to the candidates. The flyer contained an email address and instructions on how to express interest in participating in the project. The information about the project was posted at the PHC center. PHC staff gave information to eligible candidates about the intervention during physical visits. CVW staff phoned the candidates who showed interest in participating in the intervention and gave further information about the project and assessed eligibility.

The inclusion criteria to the CVW-led RPM project were age above 18 years, being fluent in the Swedish language, having access to a smart device with the iOS operating system and internet access, and having one or more of the following chronic conditions: hypertension, chronic heart failure, type 2 diabetes, chronic obstructive pulmonary disease, anxiety, and mild depression. Pregnant women and patients with other psychiatric conditions were excluded. In total, 395 participants were enrolled during the first 3 months of the project. At the time of enrollment, 153 participants gave their written consent to be contacted about further research. The project started in October 2018 and ended after 6 months at the end of March 2019.

All the participants were scheduled for introductory group information sessions at the PHC (6-10 participants per session). The CVW enrolled the participants into the platform and decided on a monitoring plan for each participant based on their chronic disease(s). An information technology assistant from the RPM platform provider helped the participants to download an iOS app into their smartphone device and paired the app to the selected noninvasive sensor(s) via Bluetooth. The participants received verbal and written instructions on how to share their vitals (blood pressure, weight, sleep rhythm, step count), track their vitals, fill out forms, receive standardized information, and communicate asynchronously by chat or synchronously via prebooked video appointments with the CVW.

The participants were able to transmit health data or respond to questionnaires without restrictions in time or place. Vitals and answers to questionnaires were transferred via the participants’ apps to a secure cloud server.

### Recruitment of Study Participants

All 153 RPM project participants who consented to be contacted were invited by the last author (MT) via email (including 1 reminder) to receive information about this qualitative study. A total of 21 (13 female) patients responded to the invitation and accepted to be interviewed. The number of participants at each process stage for the recruitment to the in-depth interviews is presented in Table 1.

**Table 1.** Process stage for in-depth interviews.

Process stage	Number of participants
In the RPM^a^ intervention	395
Agreed to be contacted for qualitative study information	153
Agreed to be interviewed	22
Completed the interview	22

a RPM: remote patient monitoring.

### Data Collection

Due to the COVID-19 pandemic, the in-depth interviews were conducted digitally via the Zoom communication platform [41] and audio-recorded after the participants’ verbal consent. Only the interviewer and the participant were present during the individual interview.

### Interview Guide

A semistructured interview guide was designed to attain an in-depth understanding of participants’ perception about the interaction with the CVW team. A pilot interview with 1 person not included in this study was performed by the first author (AJ, PhD student, M.D, specialist in family medicine, male). The final interview guide was approved by all the authors (Multimedia Appendix 1).

Between March and May 2021, 22 in-depth interviews were conducted. Each interview ranged between 45 and 90 minutes. An external research assistant (female) with previous experience in qualitative methods, without any affiliation to the participants, conducted the interviews after initial training in the semistructured interview guide and with the guidance of the last author of this study (MT, associate professor in family medicine; female).

### Data Analysis

All interviews were recorded and transcribed verbatim. Data analysis was conducted in Swedish. The principles of qualitative content analysis were conducted according to Graneheim and Lundman [42]. A basic inductive content analysis process was applied [43]. The first and last authors selected meaningful phrases and sentences independently. The second step was to condense them to meaning units, and the third step was to set open codes on the manifest level to avoid interpretation and abstraction at the coding stage. The condensing of meaning units into codes was revised during physical meetings in discussions between the first and second authors, and after agreement, a new clustering of codes was conducted if needed. This ensured a common understanding and a reduction in the number of codes before aggregating these into subcategories and categories. The strength of this method is that during the categorizations, the authors are going back to the original statements several times. After the coding and categorization described above, all the authors discussed the results and developed the overall theme (Textbox 1).

Textbox 1. **Examples of meaning units, condensed meaning units, and codes.**

**Quote**

 “I take my blood pressure a number of times a week, then I get someone who reviews these values and thinks whether it’s good or bad simply. So far, all the values have been satisfactory all the time, so it’s that you feel calm, and you know that someone is checking it.”

**Meaning unit**

 I take my blood pressure and feel reassured that someone is checking and thinking whether it is good or bad.

**Condensed meaning unit**

 I feel reassured.

**Code**

 Someone is keeping track and is watching.

### Ethical Considerations

This study was approved by the Regional Ethical Review board in Stockholm, with 2 amendments (Dnr 2018-625-31/5; 2018/1717‐32; 2020‐01890). An email with study information and a consent form was sent to the participants prior to the interview. All the participants gave written informed consent before they were scheduled for an in-depth interview, and oral informed consent was recorded verbally at the start of the interview. To ensure participant anonymity, the participants’ identification was replaced by a unique identification number. This number was used to match the participants and their transcripts and to report participant quotations in this study. The participants did not receive any compensation for being interviewed.

The first author (AJ) was clinically active at the pilot PHC 1 year prior to the launch of the project. Thus, he did not participate in the data collection to avoid bias.

## Results

### Demographics

The 22 interviewed participants had a mean age of 61.2 years (SD 11.2; range 40-78 years of age). Most (n=13, 59%) participants were women. They had predominantly 1 medical condition (n=15, 68%), where 19 (86%) of these participants had hypertension. No participant was diagnosed with more than 3 medical conditions. In comparison to the CVW-led RPM project, the 385 participants had a mean age of 58 years, with 62.3% (n=240) being female participants. The participant characteristics of this study are listed in Table 2.

**Table 2.** Participant characteristics (n=22) with 1 or more chronic conditions.

Characteristics	Participants, n (%)
Age (y)	
40‐49	4 (18)
50‐59	5 (23)
60‐69	8 (36)
70‐79	5 (23)
Sex	
Female	13 (59)
Male	9 (41)
Medical condition	
Hypertension	19 (86)
Diabetes	6 (27)
Mental disorder	3 (14)
Congestive heart failure	1 (5)
Chronic obstructive pulmonary disease	1 (5)
Number of medical conditions	
One	15 (68)
Two	5 (23)
Three	2 (9)

### Overall Theme

The following theme was constructed: “Sense of security and accessibility, but impersonal and uncoordinated” based on 5 categories: sense of security, care and self-care, accessibility, quality of care, and communication (Textbox 2).

Textbox 2. **Subcategories, categories, and themes from content analysis. CVW: centralized virtual ward; PHC: primary health care.**

**Theme: Sense of security and accessibility, but impersonal and uncoordinated.**

- Sense of security
  - Self-control
  - Care relationship between CVW and the patient
- Care and self-care
  - Care utilization at the PHC
  - Health awareness
- Accessibility
  - Care not dependent on space and time
- Quality of care
  - Basis for making health care decisions
  - Proactive care
- Communication
  - Participant communication with the CVW team
  - Lack of coordination between CVW team and other caregivers at the physical center

### Sense of Security

A higher number of transferred vitals and correspondence with the CVW at signs of deterioration strengthened the patients’ sense of security. The patients experienced that the CVW reacted promptly to deviating vital signs. Seeing that their vitals were stable induced a sense of security, which reduced their worries about a potential major medical event. Prompt actions taken by the CVW at signs of deterioration built further trust. The participants expressed that the CVW cared about them. A recurring argument for sense of security was the interaction with a trusted health care professional who gave clinical feedback:

Then you know that there is someone who cares, there is someone who sees my values every day which my regular doctor does not do. Then I feel safer, then there is a reason why I do this every morning.

[Participant 9]

The participants regarded their collected vitals as evidence when they interacted with health care professionals. They used this evidence to present their case when having a physical contact at their PHC. The clinicians’ access to collected vitals and medical records gave a historical perspective, which gave the patients an elevated sense of security as illustrated:

What is positive is that it feels like there is a history all the time that you can keep track of, what it looks like for me… it feels like there is a history to see in a completely different way, which gives security…

[Participant 12]

RPM with interactions by the CVW sheds light on the importance of feeling safe as expressed by the following participant:

Now you start to realize that it is incredibly important that you feel safe, important for your health and the feeling of… well if something happens, yes, then I know.

[Participant 13]

### Care and Self-Care

Continuous reporting of vitals gave the participants insights into their underlying condition and the symptom related to their chronic diseases. Discussing deviating vitals with the CVW enhanced their understanding about the causes of deterioration. Health awareness resulted in patients taking steps toward active lifestyle changes. Motivation for tracking vitals increased as it gave better insights about their condition, as 1 participant described:

… say that I had gone to the health centre every other month or quarterly and taken my blood pressure there, then I had not thought about the times between each visit that you may have felt bad and that it may have been because the blood pressure had been too high. Now it was very easy when you feel a little strange, this does not feel good, well then, I can take the blood pressure and then I have better understanding of how I feel right then and how it feels to have this high blood pressure.

[Participant 20]

Feedback on the collected vitals and being monitored also motivated some participants to take further steps toward active lifestyle changes, as expressed by the following participant:

Yes, but it is with this that I have started to move a lot more, it has really motivated me to do not just walking but also other physical activity like with swimming and cycling and things like that, there have really been changes there.

[Participant 3]

Insights about the correlation between lifestyle choices and the stability of vitals were generated in the dialogue between the participants and the CVW. These insights motivated the participants to change harmful lifestyle behaviors. The participants sensed that the CVW tried to improve their health outcomes by working both reactively and preventively. One participant described an aha feeling:

Just that feeling that it was nice that someone has an ambition to make it possible for me as a patient to improve. A little aha-feeling… It felt like someone cared, like “now we'll do good before it gets too bad,” so that was positive.

[Participant 1]

### Accessibility

The participants expressed that they refrained from contacting the PHC for what they perceive as bagatelle symptoms because they want other patients with greater medical needs to have access to care. This mindset might lead to deteriorating symptoms that might result in unnecessary complications. The RPM platform reduced thresholds for asking questions about their chronic condition, which enhanced the participants’ sense of accessibility to the CVW compared to the medical staff at the PHC center as expressed:

I contact healthcare when I need to. If I had a question that was important to me, but that I wouldn’t call the regular health centre about.…When I can write that easily, maybe I’ll reach out to them more often if I have questions or such…. Usually, I don’t do that; I go to the doctor when I am really unwell…

[Participant 16]

The participants were able to travel with their noninvasive sensors and contact the CVW to follow up on their vitals. This opened a new dimension of accessibility to health care for some participants who had been restricted from traveling:

A benefit is that you are not tied to sitting at home; even when I travel, I have the blood pressure monitor with me, and I can take those readings, which can also be used for monitoring.

[Participant 6]

### Quality of Care

The difference between the number of data points collected by traditional health care versus RPM via the CVW raised new perspectives. The participants reflected that the collection of additional data points could result in better decision-making by the health care staff:

The staff I have contact with have significantly better information available once I contact them.

[Participant 9]

A higher frequency of data transfer by sharing of vital measurements leads to a better basis for medical decision-making for the CVW according to patients’ experiences. They found that continuous control of vital signs allows both patients and clinicians to detect signs of deterioration. This allows clinicians to adjust treatment plans more stringently to stabilize patients’ vitals. Medical adjustment made based on only 1 measurement is not as reliable as those made based on a higher frequency of measurements according to the following participants:

…it became more quantitative data of course because it felt like the case became more, it became a better basis in the continued care…

[Participant 20]

To go to the healthcare centre and do a measurement at some point in the year does not actually say much more than what it is right then. I think it is good to have some continuous measurements, partly it is a security to get information if there is something that seems strange.

[Participant 9]

To be reminded of reporting values was expressed as feeling that “someone taps on the shoulders.” This proactiveness of the CVW was more appreciated than the traditional way of contacts from the health care center.

…if I did not leave my daily vital measures they contacted me. The healthcare centre does not do this in the same way, but it is this part that I appreciate, that there is someone who taps you on the shoulder and says that now is the time to leave some vital measures.

[Participant 19]

### Communication

This category includes the communication between the CVW, the participants, and the PHC staff. A communication plan was established for participant referrals from the CVW to the PHC. The CVW was being seen as a quite separate entity, and some participants mentioned that it was not important where the CVW was located physically. The ease of use of the app, including the CVW response through the asynchronous channel, was expressed as faster, easier, and simpler compared with the communication channels with the PHC center:

…if they are in the healthcare centre or if they are sitting elsewhere, I have no idea and I do not care so much either. It does not matter to me.

[Participant 6]

Much simpler. Much faster response, much easier…Yes, I have much more communication with the healthcare now than I had before…

[Participant 4]

The participants identified that the clinicians at the PHC did not have access to their collected RPM data. In addition, the general practitioners were not interested in getting the information regarding the collected vitals. The lack of communication and collaboration between the PHC personnel and the CVW was mentioned by some participants:

I can't quite understand if this team collaborates, or to what extent they cooperate with the healthcare centre, or if they are updating them on the values that I submit.

[Participant 19]

But I don't know if that’s the case; it doesn’t seem like it when I’ve wanted to show my values to the doctors. There was a female doctor, and she said, ‘That’s not for us.”

[Participant 5]

The asynchronous communication, in combination with the standardized automatic replies and notifications sent to the participants, was described as impersonal:

So, it feels safe although it is quite impersonal, I would say.

[Participant 22]

Yes, it’s not the same staff that I have contact with anymore; it could be random people reaching out who I don't really know who they are, so it becomes more anonymous that way…It becomes impersonal that way.

[Participant 11]

The participants noticed that there was a lack of coordination between the CVW and the PHC center. Also, some participants wished that their efforts of vital submissions would result in better transparency and utilization of health data between the patients, the CVW, and the PHC center. There was a sense of disappointment that the PHC staff did not have access to their health data through the platform and that they felt rejected when they tried to show their data to the health care personnel at the PHC center. One participant expressed lack of coordination as follows:

I lacked some natural spin-offs such as my general practitioner or others that I met with would be informed and kept track of this. But they seemed unaware that I was connected to this system.

[Participant 1]

## Discussion

### Principal Findings

The unique contribution of this study lies in exploring patients’ perspective of CVW-led RPM in a PHC setting, bringing a new dimension into the field of RPM in comparison to on-site RPM by the PHC staff. A principal finding of this study was that the participants did not mind that the CVW staff did not belong to their ordinary PHC. The qualitative data highlight the participants’ appreciation for engaged, accessible, and well-prepared CVW staff rather than an attachment to their PHC clinician. The participants experienced similar benefits of RPM as the traditional model of RPM, such as enhanced sense of security [12], improved accessibility [18], positive effects on self-care [44], better quality of care [45], and the need for improvement in communication between the patients and the staff [17,46]. In addition, this study underscores the challenge of data transfer and communication between the CVW staff and the PHC staff. The main theme of this study represents the main result: *Sense of security and accessibility, but impersonal and uncoordinated*.

### Interpretation of Findings

The participants valued the CVW-led RPM in terms of staff medical competence, availability, and prompt feedback in comparison to their regular clinical contact. This indicates that patients’ trust can be earned through virtual interactions, even with unfamiliar health care staff. In this case, the CVW was able to earn the participants’ trust by following a set of protocols to monitor incoming data to detect the signs of deterioration and reacted promptly by reaching out to the individual.

Similarly, Walker et al [17] found that RPM participants reported an increased sense of safety when clinicians responded to clinical signs of deterioration. A sense of safety is defined as an internal sense—an intrinsic state based on faith and trust in oneself and others [47]. The capacity for the RPM system to operate safely lies in the possibility of understanding a participant’s symptoms and health behaviors. A sense of safety also relates to participants’ ability to process health data and awareness that the health care professionals continuously monitored their data [12]. Although a false sense of security was not mentioned by the participants, one could argue that there is a potential risk for a false sense of security as an extended outcome of organizational and workflow challenges that arise during the introduction of new ways of working, as described in the literature [48] .

The automated response in the patient application gave a sense that someone was constantly monitoring their vitals. But the dismissal of RPM data by some individual clinicians at the PHC upon physical visits may have contributed to the participants’ experience of uncoordinated care. However, the communication gaps need further investigation.

A higher number of interactions with the CVW with a faster response rate compared to their usual care at the PHC prompted a sense of accessibility to care. Access to asynchronous communication and data visualization by the RPM application gave the participants a new perspective regarding the accessibility and continuity of care. Previous studies have described that improved accessibility increases the chance of getting medical attention [49], resulting in the avoidance of hospitalization [50-52]. Hall et al [10] also describe that patients’ access to technology can aid in the development of effective health behavior interventions. The ability to share vitals from the convenience of their residence at any time of the day resulted in the sense that they had better accessibility to the CVW than to their PHC center. By eliminating confinements of time and space, their interactions with the CVW felt more adequate since they were able to ask specific questions about their underlying health conditions. The participants sensed that the answers provided by the CVW were based on facts from the collected health data. This interpretation is consistent with prior findings that patient trust increases when health data are accurate and complete [53]. Improved accessibility also reduced the threshold for help-seeking behavior from the participants. In turn, the effects of continuous interactions between the participants and the CVW in combination with the prompt response rate from the CVW enhanced their sense of continuity of care. The combination of accessibility to care and sense of continuity of care became the driving factors for their sense of security.

Previous studies found that future care models of chronic disease management will shift toward self-care facilitated by digital technology, social support, and clinical expertise [54]. Self-care is based on long-term adaptations of lifestyle behaviors with adherence to preventive or therapeutic regimens with the potential of improving health outcomes [55]. The cornerstone in self-care is collaborative care between the patient and the health care provider and individual education in disease management [56,57]. Several studies indicate that RPM empowers patients by enhancing their motivation to adhere to the treatment plan, understanding of their chronic condition, and ability to follow their vitals [17,58]. The participants of this study described enhanced knowledge about their chronic condition, awareness and understanding of deviating vitals, and adherence to long-term lifestyle changes. The collection of vitals from the comfort of their residence enhanced the sense of measurement accuracy. In line with previous findings [59], we found that the collection of vitals is a major key for allowing patients to actively share their personal health data. By observing the longitudinal trends of their vitals, the participants gained a better understanding of the effects of their lifestyle choices, resulting in deeper insights into their chronic condition. As described by Quinn et al [60], patient-gathered health data allow the patients to better understand their disease process, monitor their symptoms, and feel connected to their health care provider. These insights, with their ability to discuss their observations with the CVW, evolved in revising their health goals, leading to self-knowledge that was used for better self-care.

Stavropoulou et al [61] describe the quality of care as “holistic care, addressing all patient needs with competency and aiming for the best patient outcomes”. The participants expressed confidence in the CVW’s ability to detect deterioration signs based on health data trends. A common notion among the participants was that a higher frequency of shared vitals resulted in a better basis for medical decision-making, resulting in higher quality of care in comparison to annual checks made at the PHC center. The participants anticipated a reduced risk of deterioration because the CVW reacted to deviated vitals and applied preventive measures for restoring the participants’ health to achieve better health outcomes. However, 1 area of consideration regarding longitudinal collection of health data at a high frequency is the ethical aspects concerning boundaries of confidentiality and the clinician’s access to vast amounts of data [62]. The participants in this study did not express concerns regarding compliance to data regulatory frameworks for protecting their data privacy.

The inclusion criteria requiring participants to have access to iOS smart devices and sufficient digital literacy have excluded less tech-savvy individuals or those from lower socioeconomic backgrounds. This selection bias potentially limits the generalizability of the findings to broader patient populations, consistent with prior research highlighting digital divides in remote health monitoring uptake [29].

This study identifies that clear organizational routines and communication strategies between patients, CVWs, and their PHC center are crucial for gaining patient confidence in a CVW-led RPM setting. Several participants expressed a sense of uncoordinated health care due to a lack of work routines for sharing information between the CVW and the PHC center. While RPM facilitates continuous data collection and clinical oversight, our findings highlight persistent communication gaps between the CVW and patients’ primary care providers, which may hinder integrated care delivery. Addressing these collaborative barriers is critical to realizing the full potential of CVW-led RPM. Setting clear goal-oriented communication strategy between caregivers and patients can increase the success rate for establishing RPM [63,64]. This finding might also be relevant to setting up a CVW-led RPM within PHC settings. Our participants appreciated the ability to communicate with the CVW without being confined to the opening hours of the PHC center. Although they appreciated the better accessibility to the CVW, they argued that the asynchronous way of communication felt impersonal. This impersonal sentiment can be due to the transactional nature of digital care with fewer nonverbal cues and limited opportunities for nuanced empathy that can hinder traditional elements of small talk, creating a sense of anonymity and the decreased opportunities for emotional and social support in comparison to standard clinical visits. Despite this limitation, enhanced patient agency, transparency, and collaborative goal-setting, all supported by CWV-led RPM, can foster a sense of partnership and engagement, thereby mitigating feelings of isolation.

Previous studies describe that an increased number of communications require new work routines for the health care personnel [58] and that there is a continuous need for improvement in the communication between the patients and the staff [17,46]. Our findings suggest that clear communication strategies and work routines could reduce the perception of impersonal interactions from the participants’ perspective.

Despite advancements in RPM technology after the COVID-19 pandemic, there are still barriers for scalability of RPM in PHC settings [19], making the findings of this study relevant to understanding these challenges and potentials of CVW-led RPM.

### Strengths and Limitations

To support trustworthiness [42], we described each phase of our study methodology from data collection, analysis of data, and interpretation. The credibility [42] of the results has been validated by internal considerations regarding the reliability and accuracy in every process of this study. To improve the reliability of the study data, clear coding rules were applied so that each researcher could independently interpret the data. The trustworthiness and credibility of the study were also strengthened by providing detailed contextual information, conducting member checks, and performing a pilot study before data collection.

A primary limitation of this study was the lack of patient involvement in the design process. The limitation to data collection correlates to the recruitment of participants to the RPM project where patients who lacked access to an iOS smart device were excluded. This reduced the potential number of candidates with a lower socioeconomic background. An additional limitation of this study is the lack of information about the participants’ digital literacy and experience of using iOS smart devices. However, all the participants included in this study had access to iOS smart devices, and they received instructions for how to use the RPM app prior to getting connected to the CVW for sharing their vitals. To ensure dependability [65] on data collection, the participants were required to be fluent in the Swedish language. In addition, a semistructured interview guide was used to enhance dependability. This study did not explore the importance of how the underlying disease impacted the patient’s perception of the CVW-led RPM. Such aspects need to be further investigated in further research.

Transferability [65] was assured by describing the study setting, methods, and time frames for the collection of data. Despite the fact that the pool of the participants may not represent all the participants of this RPM project, we recognize that there is a strength in the large sample size contributing to data collection. Since some participants had left the RPM project 18 months prior to being interviewed, recall bias may have been introduced. In addition, the duration of participation in the project varied among the participants, which might have affected their view and understanding of the organizational structure and the strengths and limitations of the project.

To obtain confirmability [65], the research team audited the results and discussed the analysis repeatedly. Research reflexivity was maintained through continuous dialogues within the research team regarding personal biases that might affect data interpretation. The final strength of the study material is the inclusion of various points of view from participants as well as the research team, which is required in qualitative studies.

### Conclusion

Patient perceptions of a CVW-led RPM of chronic diseases in PHC could be condensed to a “Sense of security and accessibility, but impersonal and uncoordinated,” with 5 underlying categories: sense of security, care and self-care, accessibility, quality of care, and communication. This study reveals the following learnings from a patient perspective regarding CVW-led RPM: sense of security due to the health care staffs’ prompt reaction on signs of health deterioration, improved motivation for care and self-care, sense of accessibility to health care staff, and enhanced quality of care.

Our findings suggest that by addressing patients’ needs for new organizational routines for patient-caregiver communication, RPM via centralized virtual wards can better realize the potential of this technology.

Future research is needed to explore the health care personnel’s perceptions regarding their interactions between the CVW and PHC.

## Supplementary material

10.2196/78780 Multimedia Appendix 1 Semistructured interview guide (translated from Swedish).

## Abbreviations

CVW: centralized virtual ward
PHC: primary health care
RPM: remote patient monitoring
