# Supplementary material for: Patient’s Perceptions of a Centralized Virtual Ward for Remote Patient Monitoring in Primary Care: Qualitative Study
Source: JMIR Hum Factors. 2025 Dec 1;12:e78780. doi: 10.2196/78780 (PMC12670054; doi:10.2196/78780)
Supplement: Multimedia Appendix 1 [file humanfactors-v12-e78780-s001.pdf]

Appendix

Supplement 1: Semi-structured interview guide (translated from Swedish)

**Category**	**Question**
Introduction	• Gender (need not be asked but more noted)<br>• Age<br>• Do you have any of these diseases: COPD, Heart Failure, Type II Diabetes, High Blood Pressure?<br>• How long did you use the service?<br>• Are you still using the service today?
Use	• Can you try to explain what kind of service you perceive that Capio Hemma is?<br>• Describe how you joined Capio Hemma?<br>• Have you experienced difficulties and challenges using “Capio Hemma”? Can you further develop your answer? Feel free to give examples!<br>• Have you experienced benefits of using "Capio Hemma"? Can you further develop your answer. Give concrete examples!
Structure	• Have the staff at Capio Hemma influenced your role as a patient and the role of the care staff and your relationships with each other? Can you please elaborate?
Process	• Can you describe Capio Hemma's care team's way of working? (follow-up question: evaluate the difference between the primary care center, in what way is it different, roles?)<br>• Do you feel that “Capio Hemma” has affected your care? If yes, can you describe in what way has Capio Hemma affected your care?<br>(Example on follow-up questions to ask:<br>a. The availability of your healthcare<br>b. The communication between you and the healthcare<br>c. your trust in healthcare<br>d. motivated you to make lifestyle changes.<br>e. your ability to participate in decisions about your treatment and health care.<br>• How do you feel that you receive support in your self-care via Capio Hemma's care team compared to regular healthcare?<br>• How do you experience the collaboration between the primary care center and the care team that monitored you?<br>• How do you reason about your contact paths with healthcare when you have access to Capio at Hemma?
Result	• What were your expectations when you joined Capio Hemma?<br>In your opinion, what effects has Capio Hemma had on your:<br>a. Investigation and diagnostics?<br>b. Treatment?<br>c. Health and preventive health care?<br>• Describe how Capio Hemma's care team has affected your knowledge of your health and illness?<br>• Describe if and if so how “Capio Hemma's care team” affects your safety and security regarding your health?<br>• Would you recommend Capio Hemma to anyone else in your situation? If so, why? If not, why not?
Other	What is Capio Hemma?
